# Smart Stimuli-Responsive Liposomal Nanohybrid Systems: A Critical Review of Theranostic Behavior in Cancer

**DOI:** 10.3390/pharmaceutics13030355

**Published:** 2021-03-08

**Authors:** Jana K. Alwattar, Amina T. Mneimneh, Kawthar K. Abla, Mohammed M. Mehanna, Ahmed N. Allam

**Affiliations:** 1 Department of Pharmaceutical Technology, Faculty of Pharmacy, Beirut Arab University, Beirut 11072809, Lebanon; jalwattar@student.bau.edu.Ib (J.K.A.); a.mneimneh@bau.edu.Ib (A.T.M.); k.abla@bau.edu.Ib (K.K.A.); 2 Department of Industrial Pharmacy, Faculty of Pharmacy, Alexandria University, Alexandria 21521, Egypt; 3 Department of Pharmaceutics, Faculty of Pharmacy, Alexandria University, Alexandria 21521, Egypt

**Keywords:** cancer, liposomes, metallic nanocarriers, nanohybrid systems, theranostic delivery

## Abstract

The epoch of nanotechnology has authorized novel investigation strategies in the area of drug delivery. Liposomes are attractive biomimetic nanocarriers characterized by their biocompatibility, high loading capacity, and their ability to reduce encapsulated drug toxicity. Nevertheless, various limitations including physical instability, lack of site specificity, and low targeting abilities have impeded the use of solo liposomes. Metal nanocarriers are emerging moieties that can enhance the therapeutic activity of many drugs with improved release and targeted potential, yet numerous barriers, such as colloidal instability, cellular toxicity, and poor cellular uptake, restrain their applicability in vivo. The empire of nanohybrid systems has shelled to overcome these curbs and to combine the criteria of liposomes and metal nanocarriers for successful theranostic delivery. Metallic moieties can be embedded or functionalized on the liposomal systems. The current review sheds light on different liposomal-metal nanohybrid systems that were designed as cellular bearers for therapeutic agents, delivering them to their targeted terminus to combat one of the most widely recognized diseases, cancer.

## 1. Introduction

Cancer ranks as a primary cause of death and a vital barricade to elevate life probability worldwide [1]. According to estimates from the (World Health Organization (WHO), Geneva, Switzerland), 19.3 million new cancer cases and almost 10.0 million cancer deaths occurred in 2020 [2]. Cancer is a pathophysiological disorder where abnormal cells divide without control and can spread to neighboring tissues [3]. The chief aim of cancer treatment is to accomplish a cure and secondly to prolong life and relieve sufferings; hence, different treatment modalities have been utilized including surgery, chemotherapy, photothermal, immuno, photodynamic, radiation, gene, and hormonal therapies [4]. The evolution of these treatment modalities is based on the selectivity approached by these systems to conserve the viability and functionality of normal cells and direct all efforts to destroy cancer cells. To achieve such goals, the focus was directed to the development of novel nano technological drug-delivery carriers [5,6].

Nanotechnology offers an adaptable platform of biocompatible and biodegradable systems capable of delivering conventional chemotherapeutic agents in vivo, improving their bioavailability and accumulation around tumor tissues, thus enhancing their release profile [7]. Nanomaterials are small in size (1–100 nm), with peculiar physicochemical properties owing to their size and the high surface-to-volume ratio [8]. Some of these nanomaterials have reached clinical applications, and consequently, the nanomedicine field has been emerged [9,10]. Nanomedicine-based strategies have created many new horizons for clinicians as well as biomedical engineers for the prevention, prognosis, and treatment of significant diseases, mainly cancer [11]. These strategies will be able to provide the best personalized targeted drug delivery therapies for cancer patients through combining multiple disciplines to get the best outcome: treatment, imagining, and diagnosis [12,13]. From the different carrier systems investigated, lipid-based delivery systems have been extensively inspected on both the therapeutic and diagnostic levels. Lipid-based systems include liposomes, solid lipid nanoparticles, nanostructured lipid carriers, and lipid-drug conjugates, in addition to the amphiphilic micelles [14,15].

Nanoliposomes are spherical lipid-bilayer vesicles in the nanometer range; they are self-assembled, biodegradable, and biocompatible [16]. This system represents a pharmaceutically attractive carrier as it enables the entrapment of both hydrophobic and hydrophilic active moieties to augment their stability and/or solubility [17]. Additionally, it can lessen drug toxicities and upsurge their tumor uptake via extravasation through improved permeability and retention [18,19]. However, the slow passive targeting and dependency of therapeutic efficacy on the tumor type hinder the optimal therapeutic and diagnostic capacity of liposomes [20]; consequently, this necessitates the aid of other systems such as metal complexes to achieve their potential in cancer management.

The therapeutic perspective of metal and metallic complexes dates back to ancient times [21]. Metals can alter the pharmacological properties of known drugs, resulting in prodrugs with different physical properties and pharmacological effects and sometimes better drug release at the targeted location [22]. Metals include gold, silver, platinum, carbon, iron, zinc, titanium, and gadolinium. Due to their physical and chemical properties, metal nanoparticles have been applied in a wider range of biomedical applications, such as probing and labeling [23]. Therefore, metallic nanoparticles have emerged for cancer treatment to enhance therapeutic efficacy through site specificity and efficient drug tracking [24]. However, particle instability, hydrophobicity, safety profile, and synthesis strain limit their clinical translation [23].

To address the restrictions of liposomal and metal nanoparticles, a new generation of therapeutically active carriers known as liposomal-metallic hybrid nanoparticles has been established. This hybrid system is advantageous due to long circulation time, controlled drug release, targeting, and specificity [25]. Furthermore, it is capable of encapsulating drugs to provide stability in biological fluids, co-encapsulate imaging, and therapeutic agents, and improve the biocompatibility of certain polymers [26,27].

Nanohybrid liposomal-metal systems are an opportunity in the advance of multipurpose drug-delivery platforms. Even though existing cancer nanomedicines enhanced tumor accumulation and reduced drug side effects, there are still many concerns that should be endeavored before they can be considered for clinical applications. The suitable screening of the hybrid system components is essential for a novel structure that avoids drug leakage, provides high encapsulation efficiency, and maintains favorable pharmacokinetics [28]. Moreover, these systems facilitate the co-delivery of chemotherapeutics, as the combined cancer therapy proved to be effective to overcome drug resistance of specific cancer cells. Theranostic nanohybrid liposomal-metal structures provide an operative chemotherapeutic regimen through targeted and non-targeted pathways where each is achieved by diverse mechanisms, leading to more proficient treatment outcomes.

In this review, an updated overview of the role of nanohybrid liposomal-metallic systems in cancer therapy, as well as, recent novelties and applications of the aforementioned system is provided. The paper sheds light on the different mechanisms of liposomal-metal systems, namely, targeted and non-targeted carriers. Nanohybrid liposomal-metal systems theranostic applications in cancer research are the core of the article.

## 2. Targeted Mechanisms for Nanohybrid Liposomal-Metallic Systems

The term “targeted therapy” itself refers to a new generation of anticancer agents or mechanisms intended to impede a specific molecular target that is responsible for tumor growth or progression [29]. To identify the appropriate targets, a thorough investigation of the molecular changes underlying cancer ought to be achieved. The success of the therapy depends on the targeted release of therapeutics at the disease site while minimizing the off-target side effects caused to normal tissues [30]. Targeted therapy may extend to block cancerous cell proliferation, endorse cell-cycle regulation, encourage apoptosis or autophagy, use monoclonal antibodies, destroy cells by toxic substances, and often be involved with a union with other chemotherapies [31]. These smart therapies for targeted delivery are designed to be responsive to a range of different stimuli, which can either be external (light of different wavelengths and magnetic fields) or internal (pH and redox) (as for temperature, it can work internally or externally) [32]. Besides stimuli, targeted delivery can achieve their anticipated pathologic cell specificity when linked to therapeutic, imaging, or targeting agents to deliver the attached cargo selectively to the pathologic cell [33].

### 2.1. Stimuli-Responsive Systems

#### 2.1.1. Exogenous Stimuli-Responsive Systems

Exogenous stimuli-responsive delivery systems precisely control the release of drugs by external factors to overcome interpatient variability that affects drug pharmacokinetics. Different external stimuli can be used such as light, temperature, and magnetic field [34].

##### Light-Responsive Systems

Light as an external stimulus for drug-delivery systems is dominant for many reasons, such as its noninvasive nature, temporal control, and high spatial resolution, in addition to its convenience and ease of use [35]. Thus, nano-preparations that are responsive to light stimulus have been intensively investigated as they can distantly control drug delivery upon the presence of different wavelengths, either ultraviolet (UV) or near-infrared (NIR) [36].

##### Ultra Violet-Visible Stimulation

Ultraviolet-visible light has been employed in drug delivery because of its ease of manipulation, versatility, and its ability to induce chemical modification on the therapeutic nanocarriers. Nevertheless, the photo regulated drug release is not always accomplished by UV light due to shallow tissue penetration with depths less than 1 mm and may cause cellular damage, which is why it is mostly replaced by NIR as mentioned later [37]. Table 1 summarizes the studies where UV light was employed to improve the characteristics of liposomal nanohybrid systems.

##### Near-Infrared Light

Near-infrared light at a wavelength ranging between 650 to 950 nm is characterized by negligible phototoxicity and deep penetration into biological tissues (1–3.5 mm) [41]. NIR light can be converted with suitable optical materials into heat for photothermal therapy (PTT) and reactive oxygen species (ROS) in photodynamic therapy (PDT). Photothermal responsive smart delivery systems achieve on-demand drug release through nanomaterial-mediated PTT upon NIR laser irradiation to generate heat that destroys targeted cells. Similarly, PDT exploits photosensitizers and light irradiation to generate ROS that induces cancer cell death. Eventually, it is possible to combine thermal and ROS sensitive components into nanohybrid liposomal-metallic systems to achieve photoresponsive drug release for specific cancer therapy [42].

Gold nanoshells loaded with doxorubicin (Dox) in hollow silica core shells employing nanoliposomes as a template were prepared by Wu et al. [43]. After doxorubicin-loaded liposomal/silica/gold nanohybrid being irradiated, the absorption peak intensities were higher than those obtained from the non-irradiated nanohybrid system, indicating a rapid release of the drug under the influence of NIR light. Moreover, doxorubicin-loaded liposomal/silica/gold nanohybrid accompanied by light irradiation caused the maximum toxicity to human liver cancer cells. Doxorubicin-loaded liposomal/silica/gold nanoshells possessed a synergistic effect on the cell growth rate due to dual therapy (chemo- and photo-thermal). Nevertheless, in vivo studies and the long-term use of hollow gold nanoshell safety required a rigorous examination. With this new approach, cancerous cells can be synergistically targeted by combining liposomes and energy-absorbing particles to facilitate the cellular uptake of many macromolecules [43]. Hashemi et al. aimed in their study to enhance the bioavailability and pharmacokinetics of nanoparticles intended for tumor-targeted delivery by combining the advantages of liposomes with a layer-by-layer synthesis method (LBL). Different layers of graphene oxide (GO) and graphene oxide conjugated poly (L-lysine) (GO-PLL) was deposited on the surface of cationic liposomes, (LBL Lipo-graph) to encapsulate doxorubicin by electrostatic interaction [44]. The number of layers formed during LBL construction and GO or GO-PLL to liposome mass ratio effect on zeta potential and the average size were studied (Table 2). As the mass ratio increased, zeta potential decreased while the average size increased. More number of layers made the coat difficult to be constructed, and the effective LBL formation depended on GO and GO-PLL concentrations. The infrared response suggested that four layers are sufficient to induce a gel-to-liquid phase transition in response to NIR stimulus. The optimal LBL Lipo-graph had a particle size of 267.9 ± 13 nm, a zeta potential of +43.9 ± 6.9 mV, and encapsulation efficiency of 86.4 ± 4.7%. Dox release displayed a biphasic release profile with an initial burst from liposomes (22.48%) which was faster than that from LBL Lipo-graph (17.85%) in the neutral medium for the first 8 h followed by a sustained release over 16 h. This faster release was elucidated by the hydrophobicity of Dox and the short diffusion barrier thickness. The release profiles of Dox from the nanoparticles were pH-dependent as it was higher in both LBL Lipo-graph and bare liposomes in an acidic medium. This dependency was related to the protonation of phosphate salt by pH shifts from 7.4 to 5.2. The antitumor effects of LBL Lipo-graph with and without NIR laser irradiation were further examined on MD-MB-231 cell lines. The four LBL Lipo-graph encapsulating Dox exerted dual-mode targeting, where NIR light was absorbed by GO and GO-PLL shells and converted to heat to activate a gel-to-liquid phase transition of the liposomal membrane leading to release of Dox into targeted tumor cells, additionally, the light absorbed provided photothermal effect on the cancer cells. This system has potential for the co-delivery of multiple therapeutic agents for dual chemo-photothermal treatment of cancer.

Similar to Fritze et al., Dox release from liposome was pH-dependent and showed a maximum increase at pH 5.5 [45]. The presence of a shielding shell around the liposomes triggered Dox release from both formulations slower than from LBL Lipo-graph, due to a decrease in drug diffusion rate. A mutual path was followed by Gui et al. where gold nanoclusters (AuNCs) and doxorubicin embedded in the internal aqueous cavity of the photosensitive liposome were prepared by supercritical CO_2_ method for a photothermal triggered drug release and tumor therapy [46]. Light irradiation of AuNCs/Dox liposomes elevated the temperature of the media, which is essential for selective tumor treatment. On the contrary, blank liposomes did not reveal any temperature change. The cellular uptake of the nanoparticle system into the tumor HeLa L929 and HepG2 cells was through endocytosis. After irradiation for 6 min, the temperature increased to 48 °C, and Dox was rapidly released with an abrupt increase in its cellular concentration. The release pattern confirmed that the photothermal effect induced a phase transition rather than complete structural damage, where the structural damage would lead to the continuation of Dox release even after cooling. The results proved that the developed system functioned as an on–off switch for drug release through controlled irradiation time and intensity [46].

Black phosphorus (BP) is a biodegradable 2D nanomaterial with good potentials in photodynamic therapy, drug delivery, bioimaging, and photothermal therapy [47]. BP quantum dots (BPQDs) were embedded in the hydrophobic bilayer of the liposomes by the thin film hydration method, and the aqueous core was used to load the therapeutic agent [48]. Dox was encapsulated in the system via transmembrane pH gradient technique (encapsulation efficiency 89.6%) with intent to explore NIR controlled-release effect. Dox exhibited burst release upon NIR stimulus for 5 min followed by sustained release afterward. Increasing light intensity from 0.5 to 1.5 W/cm^2^ increased Dox release from 20 to 48.1%, respectively. The released rate was highly dependent on the light stimulus: it terminates when the light is off, and it restarts when the light is back on. The in vitro intercellular studies on MCF7 cell lines in the dark and with NIR stimulus were studied. The application of NIR solely on MCF-7 cell lines led to negligible cell death (3%) suggesting that NIR has little influence on cell viability, while BPQDs liposomes caused 29% cell death in the dark, and the same concentration of BPQDs-liposomes/Dox system led to up scaled the killing effect to 91% after application of light irradiation for 10 min. The improved efficacy of the chemo-photothermal therapy system was derived from the rapid Dox release and the photothermal impact which improved the cellular permeability thus enhancing the extracellular accumulation of Dox [48]. However, the author lacked the in vivo animal model investigation which could provide more solid evidence for both the system safety and controlled-release capacity.

##### Temperature-Responsive System upon NIR Stimulation

Temperature-dependent drug delivery proposes a great perspective due to their flexibility in design, phase transition temperatures, passive targeting, and in situ phase transitions. These systems can function in the normal physiological state along with the tumor-targeted hyperthermia (42 °C) [49]. Dox was encapsulated into gold nanoparticles (GNPs) or hollow gold nanoparticles (HGNPs) by pH gradient and then were embedded inside thermosensitive liposomes (TL) where the GNPs and HGNPs acted as a “nanoswitch” to killing tumor cells upon NIR stimulus, by hyperthermia and triggering Dox release [50]. An initial burst release (80%) of Dox from HGNPs after 1 h at 42 °C and was less than 10% at 37 °C, where liposomal formulation increased the release rates at regional hyperthermic temperatures. Upon NIR exposure for 30 min, a boost in drug release (83%) was observed. The photothermal transduction ability of HGNPs inside TLs elevated the temperature above phase transition temperature resulting in the triggered release of Dox. The co-delivery of HGNPs and Dox-TLs created a synergistic cytotoxicity response, as it enhanced anticancer efficacy eight-fold, increasing the survival time with 40% viability for MCF-7 cells compared to GNPs and Dox-TLs. These results were owed to the strong photothermal alteration ability of HGNPs inside the liposome and Dox triggered release. The in vivo antitumor activity study showed that after 30 days, Dox HGNPs-TL combined with NIR suppressed tumor size to 18.8% compared to 52.14% in the free drug. The dual strategy of chemotherapy and photothermal therapy decreased systemic toxicity with significant therapeutic potential for cancer treatment. Following this, Mathiyazhakan et al. reported the loading of Dox in gold nanostars (Au NSs) containing liposomes. Dox-AuNS-liposomes was exposed to NIR laser, where AuNSs absorbed this energy and within only 10 s, induced complete release of liposomal encapsulated Dox, while the cargo without AuNSs released only 10% of Dox load [51]. The therapeutic effectiveness of this complex was tested on B16 F10 melanoma cells. AuNSs-liposomes were nontoxic without laser exposure, and upon NIR exposure and transient heating of the liposomes, AuNSs-liposomes, and Dox-AuNSs-liposomes cytotoxicities induced 82.42 ± 5.23% and 93.75 ± 5.37% cell death, respectively. NIR exposure disrupts liposomes architecture and triggers anticancer agents’ release which creates an efficient platform for targeted controlled drug delivery and contrast media. This dual effect was evaluated by Ou et al. who synthesized multibranched gold nanoantennas (MGNs) by one-step seedless growth mechanism to mediate photothermal heating upon NIR light with low-temperature sensitive liposomes (LTSLs) for the delivery of Dox to MDA-MB-231 triple-negative breast cancer cells (TNBC) [52]. MGNs were able to convert the NIR light to heat to control Dox release from LTSLs at a phase transition temperature of 42 °C. At 42 °C, dox release was 90% within 5 min, while at 37 °C, less than 5% was leaked from liposomes, indicating the importance of the hyperthermia-triggered release to enhance the chemotherapy with minimal damage to healthy tissues. The hyperthermia induced by MGNs successfully delivered Dox intracellularly with 33% cell death even at low concentration (0.5 μg/mL), while at that concentration, free Dox showed only 17% cellular death. Even though in vivo assessment for these gold nanoantennas was absent, the results still validate the efficacy of the photothermal–chemotherapy combination for the controlled targeted release of Dox with negligible normal cell toxicity.

**Figure 1:**
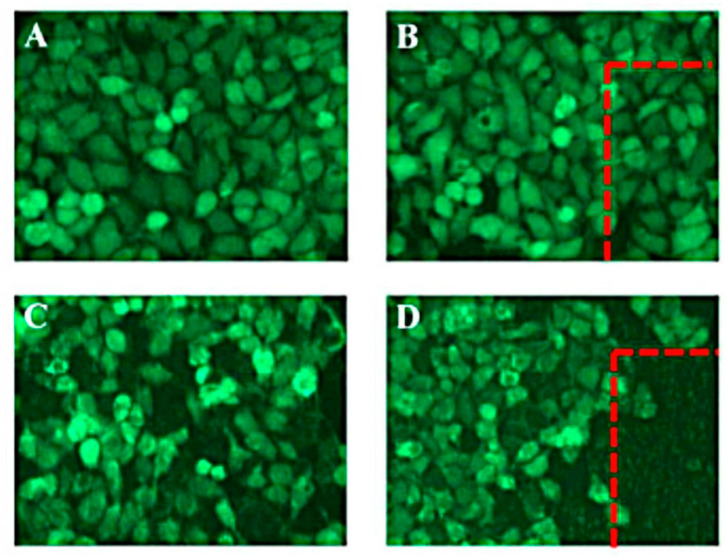
Images of fluorescence microscope of HeLa cells: (**A**) control; (**B**) cells subjected to laser irradiation only; (**C**) cells subjected to resveratrol loaded nanohybrid system with no irradiation; and (**D**) cells subjected to resveratrol-loaded nanohybrid with laser irradiation. This image was reproduced with permission from [53]. Copyright©, 2017, Royal Society of Chemistry.

Taking up another side of gold, a multifunctional responsive system loaded with resveratrol (Res) in chitosan modified thermoresponsive liposomes were coated by gold nanoshells (GNShs) [53]. The prepared system possessed broad near-infrared (NIR) absorbance and photothermal stability since the nanoparticles were stable against laser-induced photothermal hyperthermia. Resveratrol-triggered release upon NIR stimulus was investigated at pH 5 and 7.4 to mimic cancer tissues (acidic medium) and normal tissues (neutral medium). NIR irradiation for 5 min intervals within 24 h promoted 57.6% of Res release at pH 5.0 and only a 20.5% release amount at pH 7.4. The higher release at acidic medium is attributed to the presence of -NH2 groups in chitosan molecules that were protonated in acidic surroundings then Res molecules easily diffused out from the liposomes. Without NIR irradiation, the release of Res from the nanohybrid system was slow (5.7% at pH 7.4) and (13.7% at pH 5.0) during one h, and when NIR light was turned off, the drug release rate returned to its regular slow rate. Furthermore, it was found that under NIR irradiation enhanced intracellular fluorescence was visual after 1 min of irradiation in comparison with those without irradiation (Figure 1).

NIR light-induced more tumor cells killing at different tested concentrations compared to the chemotherapy (free Res) or the photothermal therapy (GNSHs) alone. NIR allowed GNSHs to absorb photon energy and convert it into heat, the elevated temperature endorsed cellular metabolism, membrane permeability, induced protein denaturation, disruption of the lysosomal membrane to release the lysosome enzymes, and induced phase transition of liposome for conducive release of Res. This novel nanohybrid liposomal-metallic system did not only displayed dual chemotherapy and PTT but also pH and NIR light multiresponsive drug release. Similarly, gold nanoshell-coated betulinic acid liposomes (AuNS-BA-Lips) mediated by glutathione were prepared by Liu et al. [54]. Gold nanoshells absorbed NIR radiation and transformed the light into localized heat to control the drug release. Analogous to what was mentioned previously, the uptake by HeLa cells was higher with NIR exposed AuNShs-BA-Lips with 83.02% tumor inhibition, representing the noteworthy synergistic effect of combined thermal and chemotherapies for multifunctional antitumor drugs. The behavior of thermally sensitive liposomes when conjugated with gold/silver (Au/Ag) core/shell nanoparticles was assessed in a study performed by Zhu et al. [55]. Loading Dox into the liposomal aqueous core through pH gradient texchnique resulted in high encapsulation efficiency (>90%). The thermally sensitive liposomes had a phase pretransition temperature around 35 °C and main-transition temperatures about 41 °C. The in vitro testing displayed that both liposomes and nanohybrid systems possess similar temperature-dependent characteristics since Dox was released from both formulations only at elevated temperature (43 °C). This observation could be interpreted, according to the authors, based on the increase in bilayer permeability at this high temperature. Moreover, a comparative trial was carried out where the authors monitored Dox release from liposomes and nanohybrids upon laser stimulus and observed that only nanohybrid formulation was able to exhibit drug leakage by remote laser illumination [56]. Cell experiments revealed that the nanohybrid system entered SKBR3 breast cancer cells through clathrin-mediated endocytosis and was able to attach to the cell surface before reaching the acidic lysosomal medium. Moreover, when SKBR3 cells were loaded with Dox nanohybrid system they showed the highest mortality after laser treatment. Moreover, Dox was encapsulated in a lysolipid-based thermosensitive liposome modified with tri-amino acid sequence (arginine-glycine-aspartate) RGD peptide (RTSL) and conjugated on the surface of microbubble (RTSL-IMBs) to test its response toward acoustic/thermo-dual stimuli, to prove whether ultrasound (US) and laser irradiation can advance drug delivery and bioavailability [57]. The authors observed that Dox remained entrapped within the liposomes at normal body temperature (37 °C) while heating to 42 °C for a short period (6 min) initiated a rapid release of Dox from RTSLs (78.4 ± 3.2%), approving its ultrafast release kinetics of RTSLs. Afterward upon applying US irradiation for 1 min and laser irradiation for 5 min a 2.8-fold increase in the intratumoral drug accumulation at 0.5 h postinjection was observed, while it took 48 h to reach the same level of intratumoral drug accumulation without any stimulus. The cellular uptake of Dox by MCF-7 cells was highly improved in the presence of mild heat treatment. Dox was spread at the nucleus margin and all over the cytoplasm in MCF-7 cells when exposed to the US only, and with the second laser stimuli step, Dox was intensely found distributed in the nucleus. This outcome confirmed that the dual stimuli enhanced the intracellular targeted delivery of Dox. Zhang et al. results agreed with those of Geng et al. where carbon dots thermoresponsive liposomal nanohybrid system was loaded with Dox in the hydrophilic core [54]. At 37 °C, there was nonsignificant Dox release after 30 min (5%), while the amount of drug released from NIR-CDs liposomes reached almost 80% after 10 min at 42 °C, and this triggered release was achieved under 1064 nm laser irradiation. The majority of HeLa cells were killed when incubated with higher concentrations of NIR-II-CDs liposomes and upon laser irradiation, while those in the absence of laser displayed a negligible effect on cell viability, indicating the effectiveness of combining photothermal and chemotherapy in cancer treatment.

**Figure 2:**
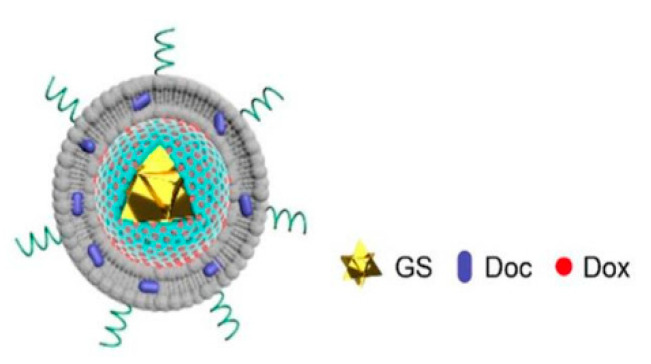
Schematic diagram of gold nanostar-liposome-entrapped mesoporous silica core/shell nanostructure. This image was reproduced with permission from [58]. Copyright ©, 2020, American Chemical Society.

From the same concept of the multimodal delivery system, Cai et al. prepared a gold nanostars-liposomes-entrapped mesoporous silica core/shell nanostructure for an “on-demand” chemo- and photo-thermal therapy [58]. Gold nanostar (GS) in the core was able to provide heat to the microenvironment, the mesoporous silica shell entrapped Dox and protected GS, and the thermoresponsive liposome layer was used to load the second anticancer agent, docetaxel (Doc) (Figure 2). When the nanohybrid system was exposed to NIR laser, gold nanostars absorbed light and converted it to heat elevating the temperature to 47 °C. This was followed by rupture of the thermosensitive liposomes, releasing both drugs. The dual chemo-photothermal therapy prompted apoptosis and revealed higher toxicity to MDA-MB-231 and MCF-7 cells. After intertumoral injection of the nanohybrid system followed by NIR, the highest tumor inhibition rate of 84.31% was observed, proving the synergistic effect of this dual system on the drug release and tumor inhibition. The prepared system was able to codeliver hydrophilic and hydrophobic drugs at the same time creating a potential platform for dual tumor treatments.

##### pH-NIR Stimulant Responsive Systems

The different pH values in tissues, tumor cells, and cancerous tissues provide the appropriate physiological stimuli for pH-responsive delivery. In tumor and cancerous cells, the pH values are lower (acidic) than those in normal tissues (neutral). This pH-dependent drug-release factor is perfect for targeting delivery for cancer and mostly referred to as “smart delivery systems”, as drug release would not accumulate at the physiological pH value of 7.4 and would take place only in the acidic environment of cancer cells [59].

Liposomes were prepared through film hydration, incorporating the chemotherapeutic agent (Dox) via pH gradient method. Gold (III) chloride (AuCl3), the metal precursor, was added into the liposomal dispersion and reacted overnight. The release study showed a relatively slow rate up to 20% in 48 h at pH value 7.4, the release increased to 60% at pH value of 5.0, and a burst release was observed upon NIR irradiation which was pH independent. This phenomenon was explained by the instability of the nanostructure membrane at low pH; moreover, the irradiation may lead to a phase transition of gel to a liquid crystalline structure which exhibited an on-demand Dox release. Higher intracellular accumulation of Dox within the nuclei after incubation of Dox/Au/lip with HeLa cell lines for 8 h reflected by a significant red fluorescence signal (Dox red fluorescence) in the nucleus. This signal was stronger after NIR irradiation; thus, hyperthermia not only promoted internalization through increasing the permeability of cells but also through triggering Dox release. The prepared nanohybrid liposomal-metal system combined with laser exhibited the highest anticancer efficacy with the lowest recorded IC50 compared to other studied systems. This excellent antitumor efficacy ought to be credited to the hyperthermia achieved by Au nanoparticles in the liposomes and the augmented release of the drug molecules. In vivo evaluation of the formulated nanohybrid system that performed in mice bearing U14 tumor, confirmed the Dox/Au/lip chemo-photothermal effect, where the NIR irradiation led to a temperature increase reaching 43 °C in only 10 s which was enough to kill tumor cells, compared to the control saline group which reached 38.2 °C. Moreover, tumor growth rates in the control groups and saline laser group were higher than other groups, while tumor inhibition rate for Dox/Au/lip plus laser was higher compared to free Dox. In addition, the absence of hepatic and nephrotoxicity was confirmed ensuring the biological safety of the system Au/Dox-Lips and Au/Dox-Lips combined with laser systems [60].

Another novel antitumor therapy combining photothermal and pH-responsive nanocarrier was investigated by Luo et al. [61]. This hybrid system consisted of gold nanoshells coated oleanolic acid (OA) liposomes (GNOLs). Chitosan was added to ensure the formation of nanoshells by connecting gold nanoparticles to liposomes. GNOLs were able to accumulate and target tumor tissue with a lower drug dose. In absence of NIR and at pH 7.4, drug release from GNOLs was slow and controlled with 39 ± 2% released after 8 h as gold nanoshells averted drug diffusion and restrained it inside liposomes, while that was rapid at pH 5.5 (53 ± 1%), indicating that GNOLs are pH-sensitive and function in an acidic environment which is advantageous for tumor targeting. The exposure to NIR significantly enhanced drug release at both pHs. This was clarified by the fact that GNOLs are capable of absorbing NIR and convert this energy into heat which caused a phase conversion in the lipid membrane. The effect of NIR was also present in vitro on 143B osteosarcoma tumor, where GNOLs inhibition rates were 73.74 ± 1.32% without NIR, and 86.91 ± 2.53% with NIR exposure with a smaller number of 143B living cells. The in vivo assessment of tumor size showed that GNOLs with NIR had the highest antitumor efficacy with a 79.65% tumor size reduction after fifteen days. This new perception of GNOLs is a promising approach for tumor treatment highlighting the importance of chemo-photothermal combined antitumor therapy.

##### Magnetic Field Stimulation

Light among the broad spectrum of nanomaterials being studied for biomedical applications, magnetic nanoparticles have captivated significant attention because of their intrinsic magnetic features, which enable them to be tracked via magnetic resonance (MR) imaging and radiology [62]. In response to the magnetic field, either alternating magnetic field (AMF) or permanent magnetic (PMF), metallic nanoparticles could generate different modalities that could be applied to trigger magnetic drug delivery, hyperthermia, and combined magnetic therapy [63]. Although clinically magnetic fields usually served as an imaging tool, it is also greatly applied in many other scenarios [64].

##### Magnetic Field-Assisted Drug Delivery

In the last few decades, magnetic-based drug delivery systems emerged as an eminent tool for site-specific targeting for many drugs. With the aid of a magnetic field, the system directs the drugs to the targeted site precisely [65]. Iron oxide nanoparticles including magnetite (Fe_3_O_4_) or its oxidized more stable form, maghemite (γ-Fe_2_O_3_), are superior to other metal oxide nanocarriers due to their biocompatibility and long-term stability, which make them the most applicable magnetic nanoparticles for biomedical applications by far [66].

Lorente et al. synthesized magnetoliposomes (MLPs) for colon cancer based on maghemite nanoparticles (γ-Fe_2_O_3_) that were coated using thin-film hydration method with phosphatidylcholine liposomes [67]. The magnetic liposomes functioned properly in the presence of a magnetic field and prompted cell migration. MLPs were tested in CCD-18 (human colon fibroblast cell line), T-84 (human colon carcinoma cell line), and in a murine macrophages cell line (RAW 264.7). Prussian blue staining of the tumor and normal cells showed intense accumulation of MLPs after 24 h in the cytoplasm, specifically in the perinuclear site and near the cell membranes. MLPs are potential theranostic tools for colon cancer and can be used in other applications due to their functionality even after internalization. Further in vivo assessments of this magnetic nanohybrid liposomal system should be studied for better correlation of the obtained in vitro results and detailed pharmacokinetic profile determination.

For additional benefits of hybrid magnetic nanoparticles and liposomes, particularly for targeting cancer cells, Jang et al. synthesized mesoporous silica core/shell magnetic nanoparticles encapsulated by liposomes (Lipo[MNP@m-SiO_2_]) with the addition of hydrophilic organic dye, Texas red (TR) for dual fluorescent imaging. To inspect the targeting efficacy of the prepared system, Her2/neu antibodies were decorated on Lipo(TR)[MNP@m-SiO_2_(FITC)] surfaces. Incubation with normal 3T3 fibroblast and SKBR-3 breast cancer cells for 24 h showed no Lipo[MNP@m-SiO_2_]-induced cytotoxicity, even at high concentrations, ensuring its biocompatibility. The nanohybrid magnetic-liposomal system was not internalized by breast cancer cells even after 6 h, until the temperature was altered from 37 to 4 °C, proving that cellular internalization was a time- and temperature-dependent process. These results supported the use of Lipo[MNP@m-SiO_2_] for targeted drug delivery in Her2/neu positive breast cancer cases; nevertheless, further work is recommended to evaluate its in vivo behavior and to monitor its therapeutic outcomes [68].

In the same manner, a biomimetic nanohybrid magneto-liposomal system has been developed as an oxaliplatin nanocarrier and evaluated for its potential activity against colon cancer [69]. The results of the study demonstrated that the nanohybrid PEGylated liposomal system enhanced the cellular uptake of the raw oxaliplatin coupled with MamC-mediated biomimetic magneto-nanoparticles. Further, coupling oxaliplatin biomimetic magneto-nanocarriers with pegylated liposomes improved its biocompatibility as hemolysis was decreased from 5 to 2%, and toxicity on white blood cells was also reduced. Liposomal hybrid revealed these several advantages without any significant reduction in oxaliplatin cytotoxic activity in colon cancer cell lines as proved by the in vitro proliferation assay. Thus, this investigation represented a great step forward in the production of a unique nanoformulation that could be utilized for local chemotherapy.

**Figure 3:**
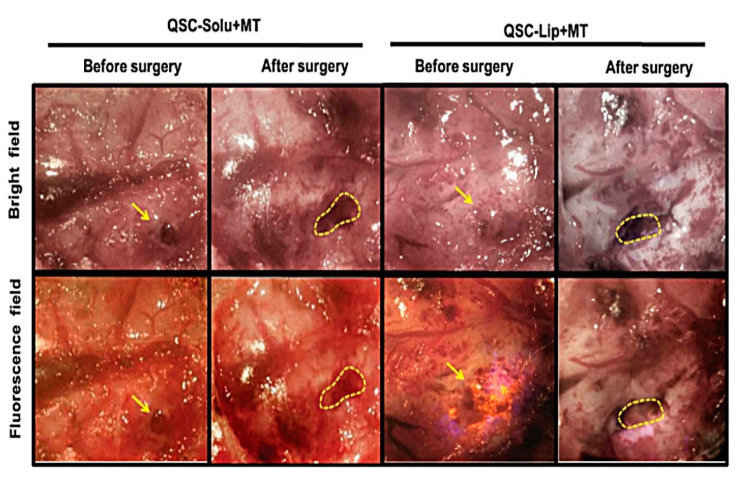
Surgical resection led by bright light and fluorescence of both; glioma in the quantum-dot–cilengitide under magnetic targeting and glioma in the quantum-dot–cilengitide–liposomal system under magnetic targeting groups, where the yellow arrow represents the glioma and the dotted lines represent the cavity after the tumor resection. This image was reproduced with permission from [70]. Copyright ©, 2018, John Wiley and Sons Ltd.

Moving to Xu et al. who developed theranostic quantum dots (QDs) liposomal system with superparamagnetic iron oxide nanoparticles (SPIONs) and cilengitide for dual-imaging-guiding cancer therapy. The formulation revealed a complete encapsulation of SPIONs and QDs in liposomes as confirmed by TEM images and good stability in several media [70]. The in vivo dual-imaging test revealed that the prepared nanohybrid liposomal-metal system not only produced a negative-contrast enhancement in glioma by magnetic resonance (MR) imaging but also induced cancer cells to emit fluorescence under magnetic field as shown in Figure 3.

Lastly, Rodrigues et al. designed a multifunctional nanohybrid liposomal-metal system containing manganese ferrite-gold core/shell nanoparticles in two forms; solid magneto-liposomes (SMLs), and aqueous magneto-liposomes (AMLs), which acted as nanovehicles for simultaneous chemotherapy to deliver the fluorescent thienopyridine derivative into the lipid membrane. In this investigation, the prepared nanohybrid systems showed diametric sizes below or around 150 nm and high encapsulation efficiencies (above 90%). Thienopyridine derivative-loaded AMLs (with the magneto-nanoparticles entrapped in liposomes) displayed a good interaction with model membranes by fusion improving its ability to transport the antitumor drug in the lipid membrane. SMLs, where the core/shell nanoparticles were covered by a lipid bilayer, showed their ability to be employed in photothermal applications as revealed by the normalized fluorescence intensity. Thus, the multifunctional nanohybrid liposomal systems containing MnFe_2_O_4_/Au core/shell nanoparticles were found to be encouraging agents for combined chemo/phototherapy.

##### Magnetic Hyperthermia

Magnetic hyperthermia is dated back to 1957 when Gilchrist et al. heated the tumor zone selectively utilizing magnetic particles in the presence of an alternating magnetic field [71]. The introduction of magnetic nanoparticles also thrust this approach into a well-researched field. Deep tissue penetration which allows for the selective killing of cancer cells without harming the surrounding healthy tissues is the main advantage of magnetic hyperthermia [72].

**Figure 4:**
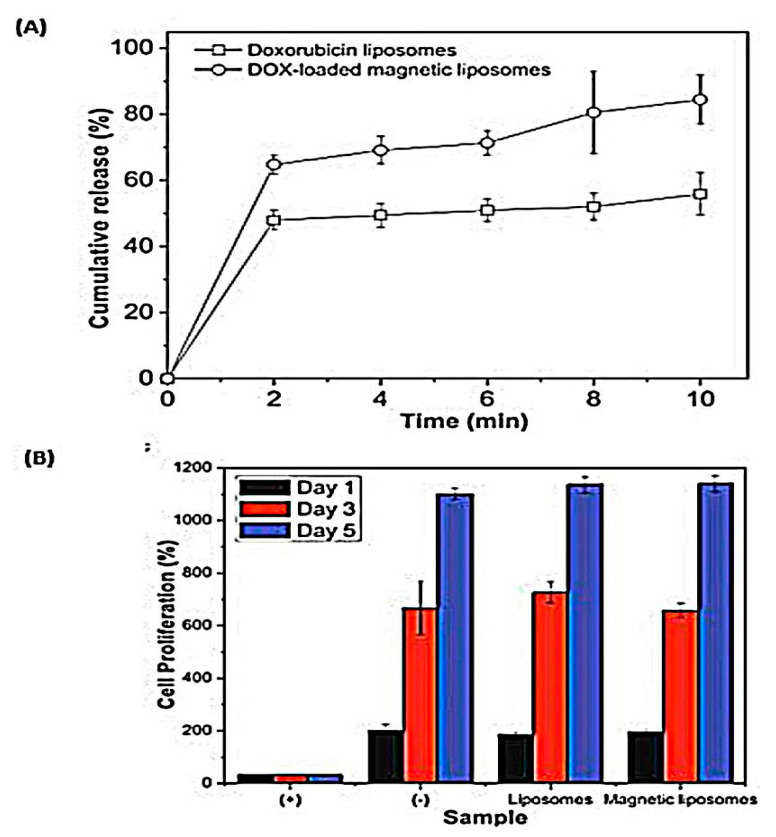
(**A**) Percentage cumulative release (%) of doxorubicin from doxorubicin (Dox)-liposomes and Dox-loaded magnetic liposomes with high field magnetic triggering. (**B**) The effect of different samples on cell proliferation (%) of lung fibroblast L-929 cells at 1, 3, and 5 days of incubation [73]. Reproduced with permission from Hardiansyah et al., Nanoscale Research Letters; published by Springer New York, 2014. CC BY 4.0 license.

Inspired by this feature, Hardiansyah et al. prepared citric-acid-coated magnetic nanoparticles (CAMNP) by the coprecipitation method, encapsulated along with doxorubicin (Dox) in liposomes by thin film hydration method; Dox and CAMNP were loaded during the hydration process [73]. Hyperthermia testing showed that temperature increased in magnetic Dox-liposomes due to the inductive heating ability of CAMNP depending on ferrites loss. In vitro Dox release under high-frequency magnetic field (HFMF) triggered a burst effect (80%) from the magnetic liposomes where insignificant burst was observed with the nonmagnetic ones (Figure 4A), parallel to what mentioned by White et al. where oxaliplatin release increased upon exposure to alternating magnetic field [74]. The authors clarified this phenomenon via the heat-induced by CAMNP which breaks up the lipid membrane inducing such a burst release. To detect the cellular cytotoxicity of free liposomes as well as magnetic liposomes on L-929 mice fibroblasts cells, MTT assay revealed that drug carriers exhibited no cellular cytotoxicity, whereas cells exposed to both liposomes and magnetic liposomes displayed a good proliferation, suggesting excellent cellular biocompatibility (Figure 4B). Colorectal cancer cells (CT-26) were utilized to investigate the in vitro cytotoxicity and the hyperthermia of the samples with 1 μm Dox concentration. After applying HFMF and within one day of incubation, CAMNP liposomes displayed 15% cancer cell death due to nanoparticles hyperthermic effect. Dox-loaded liposomes revealed a more apoptotic effect (38%), explained by the structure disruption upon HFMF application which triggered Dox release into the cells, improving its chemotherapeutic effect. Excitingly, Dox-loaded magnetic liposomes revealed the highest CT-26 cells cytotoxicity (56%). The triple-component therapy demonstrated a synergistic action of chemotherapy and hyperthermia on cancer cells.

The authors further prepared drug-loaded PEGylated magnetic liposomes by encapsulation of the oil phase of MNP and curcumin in the PEG-modified liposomes, and it was tested on MCF-7 breast cancer cells [75]. Curcumin showed a three times higher release rate upon exposure to HFMF. The cytotoxicity experiment illustrated that curcumin loaded into PEGylated magnetic liposomes could proficiently eliminate MCF-7 cells as this drug vehicle was able to adapt competently into the MCF-7 cellular compartment, stressing the potential effect of combining chemo and thermotherapies.

**Figure 5:**
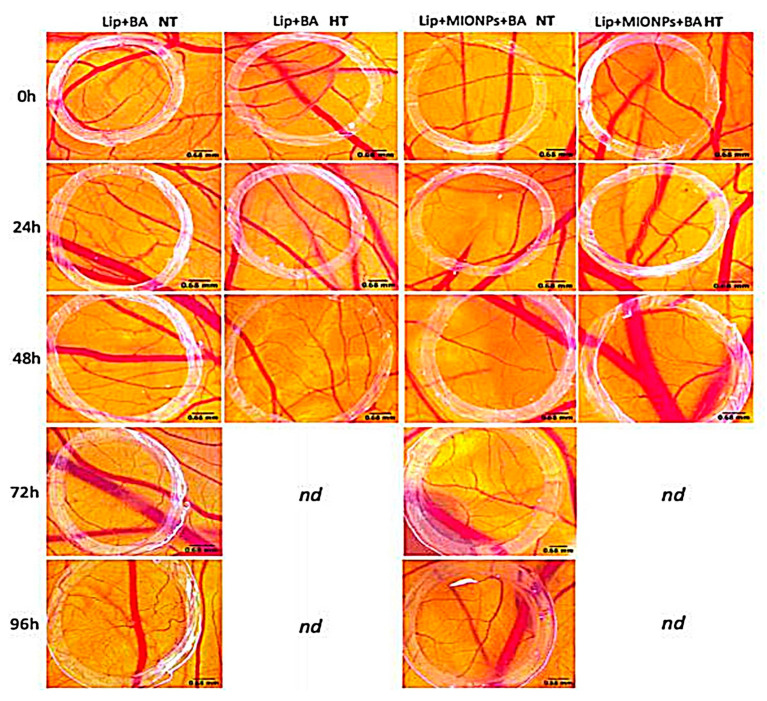
Stereo-microscopic in vivo images of the areas containing vessels treated with naked liposomes containing betulinic acid (BA) (Lip + BA) and the hybrid liposomal system (Lip + MIONPs*CA + BA) at a concentration of 25 µM, under normothermic (NT = 37 °C) and hyperthermic condition (HT = 46 °C). The specimens treated with high temperature died after 48 h; thus, they were not determined (nd) further [76]. Reproduced with permission from Farcas et al., International Journal of Nanomedicine; published by Dove medical press, 2020.

In another study performed by Farcas et al., thermosensitive betulinic acid-loaded magneto-liposomes were prepared by thin-film hydration method and assessed for their potentials in fighting human breast adenocarcinoma [76]. The findings of the current study revealed that betulinic acid (BA)-loaded magneto-liposomes could serve as a promising tool for breast adenocarcinoma therapy since the in vitro results showed that the induced hyperthermia enhanced antitumor activity when cancer cells (MDA-MB-231) were exposed to BA-loaded magneto-liposomes, while a very low level of cytotoxicity was determined by the noncancer cell line (MCF10A). Furthermore, a chick chorioallantoic membrane test was carried out to assess the antiangiogenic effect of the nanohybrid system under hyperthermia conditions. The in vivo testing endorsed the potential of the nanohybrid liposomal system in angiogenesis impairment under magnetically triggered hyperthermia conditions as shown in Figure 5.

Continuing with the magneto-liposomes platform, Cardoso et al. developed two systems: stealth (PEGylated) and non-PEGylated magneto-liposomes containing calcium-substituted magnesium ferrite nanoparticles as curcumin nanocarriers [77]. The impact of an alternating magnetic field (AMF) on curcumin release through time was studied and compared with that of the raw curcumin. The non-PEGylated nanohybrid liposomal system responded to AMF and displayed an enhanced release rate of curcumin by 1.25 times compared to the nontriggered ones. The PEGylated nanohybrid liposomal system did not respond significantly to AMF triggering but revealed an enhanced curcumin release rate (even without AMF) relative to that of raw curcumin. The developed nanohybrid liposomal systems are promising nanocarriers for curcumin in therapeutic applications, making it possible to choose nanohybrid systems with enhanced drug release (PEGylated systems) or nanohybrid systems with the triggered release by AMF (non-PEGylated systems).

##### Combined Photo/Magnetic Therapies

The developed nanohybrid liposomal systems are promising nanocarriers for curcumin in therapeutic applications, making it possible to choose nanohybrid systems with enhanced drug release (PEGylated systems) or nanohybrid systems with the triggered release by AMF (non-PEGylated systems).

A novel formulation based on photoresponsive magnetic liposomes was synthesized to ablate cancer cells [78]. The iron oxide nanoparticles were loaded within the aqueous core of the liposomes, while the lipid layer was loaded with a photosensitizer payload. This double system was translated into a dual functionality system that coupled photodynamic therapy and magnetic hyperthermia, generation of singlet oxygen under laser excitation (LE) and heat production under AMF. These nanohybrid systems directed therapeutic agents within the tumor cells, as well as the combined magnetic hyperthermia and photodynamic therapy, resulting in cancer cells apoptosis in vitro and total solid-tumor ablation in vivo rodent model. The results displayed that stimuli-triggered nanotherapies based on magnetic hyperthermia and photodynamic therapy can become attractive alternatives to conventional chemotherapy in targeting cancer cells.

#### 2.1.2. Exogenous Stimuli-Responsive Systems

Anticancer drug doses that reach the diseased cells in a controllable manner have contributed to the development of nanocarriers with a controlled release in response to high glutathione levels of cancer cells, which is being four times higher than that of normal cells. By integrating a glutathione stimulus-responsive factor into the nanoparticulate systems, the release profiles of the drugs can be targeted and improved. Thus, pH/redox responsive nanocarriers have been suggested to be good stimulant drug-delivery systems due to their ability to accumulate and internalize cancer cells [35].

**Figure 6:**
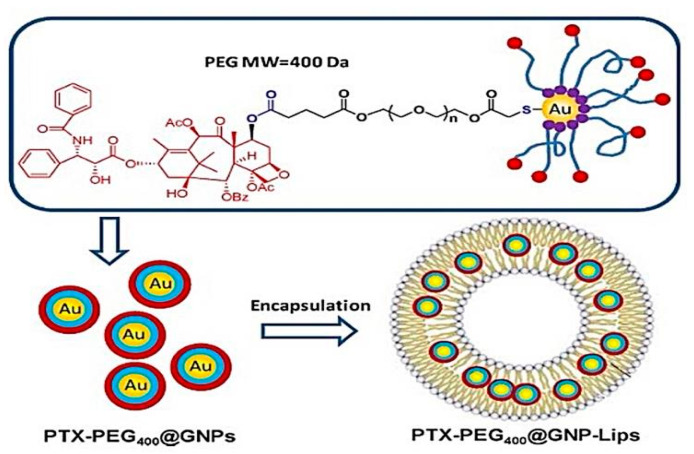
The chemical structure of paclitaxel-conjugated gold nanoparticles (PTX–PEG400@GNPs) and composition encapsulated liposomes (PTX–PEG400@GNP–Lips). This image was reproduced with permission from [80]. Copyright©, 2014, Elsevier.

A hybrid system of PEG-modified paclitaxel (PTX) conjugated GNPs encapsulated liposomes was synthesized by Zhang et al. and referred to as (PTX-PEG400@GNP Lips) [79]. Liposomes were conventionally prepared by the thin-film dispersion method. The in vitro drug release was executed via the dialysis method using GSH. At lower GSH concentrations, PTX release was faster when it was in free form than the conjugated one due to the high stability of the PTX-PEG400@GNP liposome structure. At a higher GSH level, the highest concentration of drug was released over an extended period. A cytotoxicity test was conducted on the human hepatocellular liver carcinoma cell line (HepG2 cells) which showed a paramount cell inhibition at different PTX concentrations. These findings were coherent with and completing those of Bao et al. who investigated the conjugation of mercapto-group-terminated PEG-PTX with gold nanoparticles inside the lipid carrier forming gold/hybrid nanosystem (Figure 6) intending to improve PTX physical stability and solubility [80]. In PBS at physiological pH, the system exhibited the absence of drug release; however, when the medium contained GSH, a gradual slow release of PTX-PEG-GNP conjugate from the liposomes reaching 50% after 144 h was noticed. The pharmacokinetic parameters of the PTX hybrid nanosystem in the animal model were examined. At 36 h, the PTX plasma concentration for PTX–PEG400@GNP Lip was 18.8- and 3.2-fold the values released from Taxol^®^ and PTX–PEG@GNP sodium carboxymethyl cellulose suspension, respectively. The presented values were evident by the successful release of PTX from the hybrid and ability of liposomes to protect and improve the stability of PTX thus, enhancing its circulation time. The prolonged circulation time was due to the distribution of PTX in organs, where the amount of drug distributed to the liver (at 12 h) and reached, 18.2- and 5.6-fold compared to Taxol^®^ and conjugate suspension, respectively which ensure a passive targeting to the liver. The study lacked essential experiments that could give a deeper knowledge of this novel system. Primarily, MTT assay on cancerous cell lines and normal cell lines could evaluate the cytotoxicity and safety of the formulation, compared with a free Taxol^®^ and conjugated system. Secondly, the cellular uptake and molecular assays would shed light on the ability of the developed system to uptake and the mechanism of death induced by the studied nanohybrid system.

### 2.2. Non-Stimuli-Responsive Systems

It is essential to develop strategies for anticancer drug delivery through the cell membrane and to enhance successive distribution to the biological target with high selectivity and efficacy. For this purpose, many biological ligands have been evaluated and investigated for enhancing the active targeting of the nanosystems. The ligand binds to a specific receptor on the outer surface of the target cancer cells and thus increases the cellular uptake of the active agents containing nanoparticles aiming at bioimaging or managing these cells [81]. Compared to a single ligand, an increase in the density of ligands is dominant for advancing binding and cellular uptake via the multivalent effect [82]. Different types of ligands have been utilized, such as proteins, peptides, polysaccharides, nucleic acid, and many other small molecules [81]. This section discusses the modern applications of these biological ligands in targeting nanohybrid liposomal-metal systems to cancer cells.

#### 2.2.1. Glycoproteins

Transferrin (Tf) is an iron-bounded glycoprotein that acts as an iron vehicle in the body. The presence of transferrin receptors expressed in certain cells allows targeting by transferrin-modified nanoparticles [78]. Sonali et al. exploited D-alpha-tocopheryl polyethylene glycol 1000 succinate mono-ester (TPGS) liposomes with transferrin receptor targeting as nanocarriers loaded with both docetaxel (DTX) and CdSe/ZnS quantum dots (QDs) to achieve brain delivery and imaging [83]. The highest DTX concentration in the brain was achieved with DTX-QD-TPGS-Tf, followed by the non-targeted system than commercial docetaxel (Docel^®^) with 385 ± 12.52, 99.7 ± 6.87, and 24.7 ± 2.4 ng/g, respectively, after 0.5 h of intravenous administration. The authors described such enhanced distribution of the targeted system by the augmented cellular uptake induced by liposomes, as well as the sustained drug release obtained, and the targeted delivery of transferrin that increased the blood–brain barrier (BBB) transport of DTX as an active delivery. The higher presence of transferrin receptors on BBB mediated much higher passage of liposomes via endocytosis. Targeted liposomes showed high fluorescent intensity compared to the control and non-targeted carrier, confirming the improved brain uptake and the liposomes ability to cross BBB mediated by the transferrin. These findings proved the potential application of transferrin receptor-targeted liposomes for brain tumor delivery of anticancer drugs and imaging molecules, for successful brain tumor diagnosis and treatment [83].

#### 2.2.2. Peptides

Among targeting biological ligands, peptides have many unique features viz: feasible production cost, ease of preparation, and conjugation to the surface of nanoparticles at high density, and good stability [84]. To target GPIIb-IIIa, an integrin complex found on platelets and aids platelet activation, Srinivasan et al. developed a liposomal system modified with cyclic arginine-glycine-aspartic acid peptide ligand and labeled with lipid-tethered Nanogold^®^ [85]. This study showed interesting in vitro and in vivo results, where the prepared hybrid system displayed an extensive binding to the activated platelets as revealed by the in vitro tests. Moreover, the prepared liposomal system introduced within a catheter-injured carotid artery restenosis rat model displayed excessive attachment of the nanogold-labeled cyclic arginine-glycine-aspartic acid liposomes to the activated platelets as revealed by ex vivo SEM images of the posteuthanasia artery.

**Figure 7:**
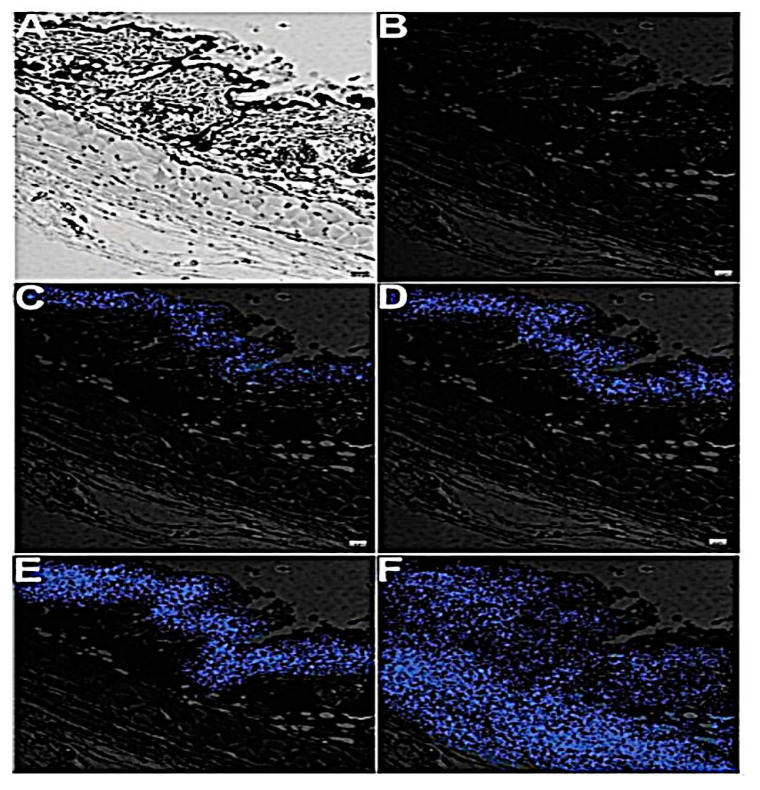
Images of in vivo mice skin permeation of carbon dots without R9 and carbon dots with R9 (**A**) the confocal laser microscopy images of untreated mouse skin in bright light and (**B**) fluorescent light. After two h (**C–E**) and four h (**D**–**F**) in mice. This image was reproduced with permission from [87]. Copyright ©, 2016, Royal Society of Chemistry. R9: peptide polyarginine. CC BY 4.0 license

The role of peptide ligand in targeting cancer cells was investigated from different aspects one of which is the transdermal drug delivery system (TDDS). As known, TDDS has emerged as an auspicious alternative approach to other routes to deliver antineoplastic agents and to avert some of the oral delivery adverse effects [86]. Patra et al. reported a generation of the cell-penetrating peptide, polyarginine (R9), and contained nanoliposomes conjugated with carbon dots (CD) for transdermal delivery of curcumin [87]. This system was fabricated by preparing liposomes using the thin-film hydration method starting from three precursors: CDs with R9 (RCDLs), CDs without R9 (CDLs), and conventional precursors (CLs) to be then loaded with curcumin. It was observed that RCDLs had the smallest particle size (128 nm), lowest PDI value (0.12), and the highest zeta potential (29.86 mV) which was linked to the presence of positively charged amino groups in the polyarginine (R9) peptide sequence. This was following the Kwon et al. study, which used cell-penetrating peptide-conjugated liposomes [88]. Moreover, unloaded-RCDLs did not exhibit any cellular toxicity toward MCF-7 cells at high concentration while the loaded curcumin-RCDLs were significantly able to kill about 90% of cancer cells. Furthermore, RCDLs displayed the highest in vitro permeation flux of the curcumin followed by CDLs and CLs through the excised abdominal mice. The confocal images of mouse skin treated with RCDLs showed that the fluorescence was detected in the epidermis and dermis of the skin within a very short period indicating that the drug had reached deeper skin layers, in contrary with the skin treated with CDLs, where the fluorescence was observed on the surface and penetrated the SC layer after some time as shown in Figure 7 [87]. This study lacks the in vivo investigation to evaluate the therapeutic efficacy of curcumin-loaded liposomal nanohybrid system to judge whether in vivo results are incoherent with the in vitro studies obtained.

#### 2.2.3. Aptamers

Aptamers are small nucleic acid (RNA or DNA) consisting of numerous nucleotides. They are small, biodegradable, and have high immunogenicity, allowing them to be a good choice as active targeting ligands [89]. Tumor-targeted liposomes containing QDs and siRNA molecules were fabricated and then coupled to anti-EGFR aptamers by Kim et al. [90]. The in vitro cellular uptake of QD-liposomes and aptamer ligand-quantum-dots-liposomal system (Apt-QD-Lipo) was assessed on MDA-MB-231 and EGFR-negative MDA-MB-453 cells and the highest transfection of QDs and si-RNA delivered to MDA-MB-231 cells cytoplasm were by the aptamer coupled liposomes. The in vivo imaging of mice treated with Apt-QD-Lipo showed high fluorescent signals indicating the location of the liposomes in the targeted tumor unlike those without coupling.

#### 2.2.4. Small Molecules

Folate receptors (FA) are known to be overexpressed in macrophages and solid tumor cells making them attractive targets for numerous nanodelivery systems through receptor-mediated endocytosis [81]. Folate-modified theranostic liposomes aimed to detect and treat hepatic cancer were formulated by Shao et al. This system was made up of HSV-TK suicide gene covalently coupling with NIR fluorescent CdSeTe/ZnS QDs [91]. Interesting in vivo results were revealed by the QD-TK-folate liposomes imaging and tissue distribution, where strong red QDs fluorescence localized in the tumor site was obtained after 0.5 h from administration and up to 7 days.

**Figure 8:**
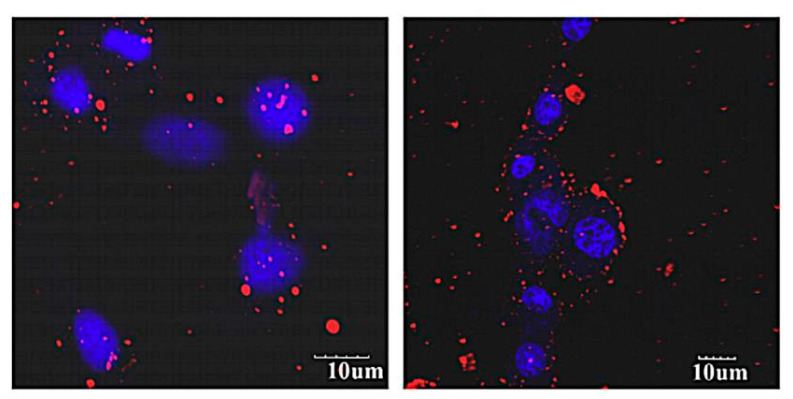
Field-emission transmission electron microscopy image of single quantum dots loaded-D-alpha-tocopheryl polyethylene glycol 1000 succinate mono-ester (TPGS)-coated liposome in a 100 nm scale. Red intensity referred to quantum dots (QDs) and blue for nuclei. This image was reproduced with permission from [92]. Copyright©, 2012, Elsevier BV.

In line with the previous study, another investigation was carried out by Muthu et al. that showed the importance of folic acid conjugated liposomes in imaging and targeting cancer cells. In this research, D-alpha-tocopheryl polyethylene glycol 1000 succinate mono-ester (TPGS)-coated folic-acid-conjugated liposomes for the co-delivery of docetaxel (DTX) and quantum dots (QDs) as a multifunctional theranostic tool for MCF-7 breast cancer cells was investigated. Confocal laser microscopy images showed higher red fluorescent intensity with the folic-acid-conjugated DTX-QDs liposomes than those non-targeted and which were more concentrated in the cytoplasm (Figure 8), confirming the efficacy of folic-acid targeting for enhancing the uptake of the liposomes by MCF-7 cells [92]. The in vitro cytotoxicity of targeted and non-targeted liposomes reflected by IC50 values was compared to commercial docetaxel (Taxotere^®^). Docetaxel-loaded targeted liposomes revealed higher cytotoxicity with IC50 (0.23 ± 0.05), while that of the non-targeted and the commercial were 1.56 ± 0.19 and 9.54 ± 0.76, respectively. The multifunctional liposomes in the current study were effective as dual agents for targeted treatment and diagnosis of breast cancer; however, additional in vivo assessment is needed for better correlation and approval of theses in vitro results.

### 2.3. Combined Targeted-Stimuli Responsive Systems

Great effort has been applied for the development of controlled drug-delivery systems aiming to improve drug therapeutic efficacy and minimize their toxicities during cancer treatment. The drug release can be stimulated by light, pH, mechanical stimuli, temperature, or biological molecules [93]. After discussing each stimulus separately, the following part investigates the effect of stimulus on the targeted nanohybrid liposomal systems to cancer cells.

**Figure 9:**
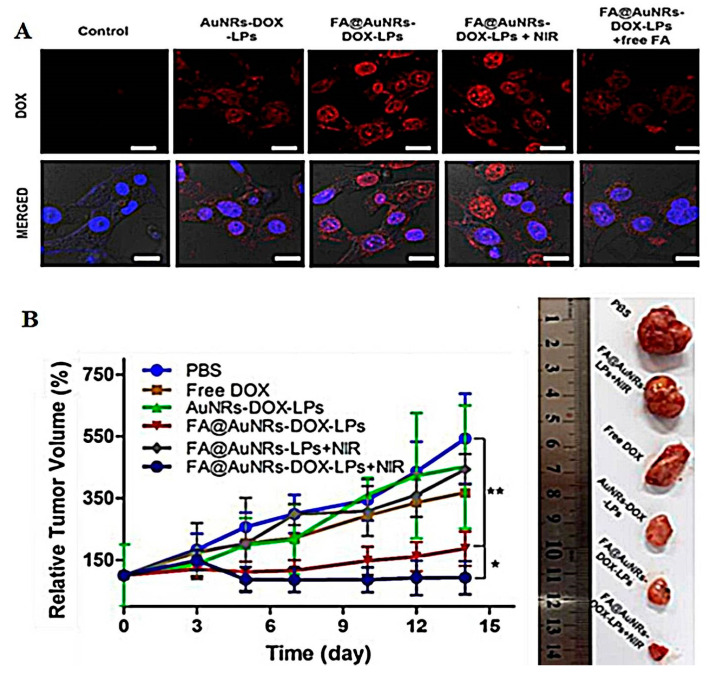
(**A**) Confocal laser scanning microscope images illustrated in vitro cellular uptake analysis of doxorubicin (represented by red signal) triggered by folic-acid-conjugated liposomal system. (**B**) Growth profiles and images of tumors of mice after gold nanorods liposomes nanocomplex injections. * *p* < 0.05; ** *p* < 0.01. This image was reproduced with permission from [94]. Copyright©, 2019, Elsevier.

A unique nanocomplex design of gold nanorods and doxorubicin in a liposomal system conjugated with folic acid was investigated by Nguyen et al. to combine photothermal-chemotherapy and target doxorubicin (Dox) to tumor cells [94]. The nanohybrid system showed a nanometric particle size of 154.32 ± 1.15 nm. The entrapment efficiency of Dox and gold nanorods inside the liposomes were 54.73 ± 2.13% and 25.12 ± 3.15%, respectively. It was obvious that the authors in this investigation depended on the ammonium sulfate gradient method for loading Dox in liposomes which resulted in a relatively low entrapment efficiency as compared to the transmembrane pH gradient method used by Alayne et al., which resulted in more than 90% loading efficiency in Pegylated liposomes [95]. Cellular uptake of free Dox and Dox-loaded liposomal nanohybrid conjugated with the folic acid system into 4T1 breast cancer cells was assessed using CLSM. It was pronounced that NIR significantly induced the uptake of drugs from the targeted liposomal nanocomplex more than the nontargeting ones as demonstrated in Figure 9A. In line with the cellular uptake analysis, MTT assay displayed that Dox-targeted liposomal system-induced significantly higher cytotoxicity with an IC50 value of 3.15 ± 0.26 μg mL^−1^ than that of the nontargeting system with an IC50 value of 4.92 ± 0.57 μgmL^−1^. Moreover, it must be noted that no cell death was observed in cell lines treated with blank nanohybrid liposomal-metal conjugate system, thus ensuring safety of the nanohybrid liposomal-metal systems. Accordant with the in vitro observation, animal experiments revealed that synergistic application of dual therapy with the folic acid targeting liposomes could effectually inhibit the tumor growth in xenograft-bearing mice as shown in Figure 9B, indicating that this platform can provide a crucial approach to target folate receptor-overexpressing tumor cells [94].

## 3. Non-targeted Mechanisms for Nanohybrid Liposomal-Metallic Systems

Tumor-distribution of the non-targeted nanocargos is largely governed by the same principles as targeted ones. Thus, not only the targeted delivery systems are capable to transport drugs into the diseases sites, but also nanocargos with no aided molecules or external stimulants are able to effectively carry drugs into the damaged cells [96]. Therefore, it is crucial to design a nanohybrid liposomal-metal system that is “maximally targeted and maximally stealth”.

In order to enhance the cellular delivery of nanoliposomes without any physical or chemical interference, Karchemski et al. suggested their attachment to carbon nanotubes (CNTs) forming CNT liposomal conjugates (CLCs) [97]. The CNTs, CLCs, and plain liposomes cytotoxicity on HEK-293 (human embryonic kidney cell lines) were compared and showed that CLCs and CNTs shared similar toxic effects even at low concentrations in contrast to the plain liposomes. Although the results obtained proved that CNTs can induce cellular cytotoxicity, the CLC can extend the role of liposomes and deliver high doses of the drug into target cells with low CNT concentration, thus resolving the related toxicity problem. Calcein was used as a model hydrophilic drug to assess the cellular uptake of the prepared formulation by HEK-293 and human fibroblast cells. The superiority of CLC over the naked liposome was confirmed by the strong fluorescent signals indicated calcein release from liposomes. The mechanism of drug loading and the enhancement of cellular uptake was not extensively discussed and requires further studies for clarification. Similar investigations tackling nanotubes are summarized in Table 3.

Metal-organic frameworks (MOFs) are hybrid unique structures, made of inorganic metal ions or clusters and organic units that represent another approach in passive delivery of nanosystems for multiple drug therapy. Illes et al. synthesized liposome-coated MOF nanoparticles composed of iron (III) and fumaric acid (MIL-88A nanoparticles), then coated with DOPC derived liposomes (Lip-MIL-88A) and efficiently loaded with irinotecan and floxuridine [102]. Coated nanoparticles with liposomes showed no leakage of fluorescent dye, unlike the uncoated carrier, proving the role of liposomes barrier formation. This result was similar to what was reported by Wuttke et al. where the dye was retained in MIL-100(Fe) nanoparticles and did not permeate through the DOPC bilayer [103]. To investigate the uptake and release of drug from this system, Lip-MIL-88A were loaded with calcein as a model drug and incubated with HeLa cells. After two days, the nanoparticles were taken up and no release of calcein was noticed, while, after three days of incubation, the release was achieved without any external stimuli. This was explicated by the fact that nanoparticles uptake occurred via endocytosis, and once they were in the lysosomal environment, the nanoparticles dissolved releasing their load. Additionally, Lip-MIL-88A was laden with SBHA, a histone deacetylase inhibitor that leads to cell death, as a model drug and also incubated with HeLa cells. The SBHA-loaded Lip-MIL-88A showed 15% cell viability after 4 days of incubation. The nanoparticles were able to safely deliver the drug to the target cells with IC50 of15 μg/mL. In addition, the authors claimed that their results concur those of Wuttke et al. regarding the intracellular uptake, where the nanoparticles were similarly taken by the cancer cells. These finding revealed that this system can be effective carrier for combination therapy especially for cancer treatment.

Gold nanoparticles (GNPs) were also applied for the non-targeted delivery of different agents. Epirubicin (EPI) is an epimer of doxorubicin with limited applications in cancer therapy due to multidrug resistance and side effects. To conquest these limitations, Kunjiappan et al. designed a passive delivery of EPI loaded in GNPs and encapsulated into the lipid bilayer of liposomes [104]. The release of EPI from liposomes was slow and steady at different pH, and the dependency of drug release on pH indicated the suitability of this system for endocytosis. The in vitro cytotoxicity of EPI-GNPs liposomes against MCF-7 cancer cell lines showed IC50 of 52% after 24 h, with amplified reactive oxygen species (ROS) levels. Treatment with this IC50 for 72 h showed that EPI-GNPs liposomes resulted in the irregular shape of MCF-7 cells with condensed nuclei, distorted membrane, and apoptotic bodies. This superior cytotoxicity was explained by the successful encapsulation of EPI in liposomes that allowed the diffusion of the drug into the nucleus, thus enhancing the therapeutic anticancer effect with minimal side effects to normal tissue, another reason for this anticancer efficacy is the generation of ROS that altered the permeability of the mitochondrial membrane, inducing apoptosis through the intrinsic pathway. Unfortunately, in vivo evaluation for the system was essential to collect elucidate pharmacokinetic and pharmacodynamics profiles.

## 4. Cancer Vaccines

A versatile nanovaccine composed of liposomes-coated gold nanocages (Lipo-AuNCs) modified with dendritic cells (DCs) was developed for the targeted codelivery of melanoma antigen peptide TRP2 and adjuvant monophosphoryl lipid A (MPLA) [105]. The presence of liposomes on the surface of AuNCs provided stability to the preparation and prevented the degradation and leakage of TRP2. The accumulation of AuNCs in the lymph nodes was utilized as real-time visualization by fluorescent and photoacoustic imaging to monitor the immunity response. The in vivo assessment of this nanovaccine against melanoma was examined in mice, and the first tumor appeared on day 10 with the formulated Lipo-AuNC and the last one on day 20, indicating that this system was able to induce an effective antitumor immune response and delay tumor incidence. The proposed nanovaccine proved to be effective in immunotherapy and in vivo monitoring of various cancer types.

Nanosystems can be carried using linker DNA resulting in controllable and reversible changes in their physical properties which offer multifunctionality. This system has been tested for drug delivery, but since most of the systems utilized are nonporous, the drug surface attachment leads to limited loading capacity, and this limitation can be overcome by the functionalization of DNA on liposomes surface. The authors reported the reversible assembly of DNA functionalized gold loaded nanohybrid liposomal system. Contrary to other studies that employed the hyperthermia property of gold to promote leakage, the distance between gold and liposomes via DNA hinders heat transfer. AuNPs were functionalized with different thiol modified DNA and achieved stability. To test the stability of the nanohybrid liposomal system, calcein was loaded as a fluorescent agent which indicated that the rigid particle assembly did not increase the liposomal leakage. The DPPC liposomes showed a time-dependent leakage, which was attributed to UV-induced liposomal damage which was further supported by the use of ascorbate as a ROS scavenger. The AuNPs assembly on liposomes completely inhibited leakage of calcien, suggesting a protective effect. The authors also studied the distance-dependent heat transfer within the nanometer scale as the AuNPs can effectively absorb radiation energy. This study presented an approximately 8 nm distance between AuNPs and liposomes through rigid DNA, and this distance allowed protection of the system form leakage due to the prevention of heat transfer from AuNP to the liposomal surface [106]. This study only investigated the properties of the developed system with the absence of molecular and in vivo evaluations.

## 5. Conclusions

The current review showed that liposomes apart from being used for the delivery of chemotherapeutic agents can also be considered as revolutionary aid for theranostic purposes. Liposomal delivery systems use has been widely accepted due to its ability to manipulate many drugs pharmacokinetics especially in cancer-targeted therapies. Additionally, metals represent an opportunity to be opportunistic to be exploited along with metal drug and metal nanoparticles as candidates for chemotherapeutic agents. Regardless of the different strategies used, and whether these nanohybrid systems were intended for targeted or active delivery, these prepared systems offered great prospects for the progress of combinational therapeutically modalities. The novel nanohybrid system builds a new pathway as an evolving system in drug delivery, yet the lack of deeply detailed in vivo performance, cellular toxicity, and metabolism, in addition to financial and quality considerations, are the main limitations for this current nanohybrid complex. Further studies will aid in the development of this system, thus facilitating their transition into clinical application.

## Figures and Tables

**Table 1:** Ultraviolet-visible light stimulus effect on the nanohybrid liposomal-metallic nanoparticle systems.

Nanohybrid Systems	Drug	Size (nm)	ζ Potential (mV)	Results	Ref.
Liposomal-gold nanoparticles	Vincristine	113.4 ± 1.7	−11.3 ± 2.2	- Enhance liposome cytotoxicity and induce apoptosis	[38]
Liposomal-gold nanoparticles	-	~200 nm	~25 mV	- Increase membrane fluidity of liposomes bilayer	[39]
Liposomal-gold nanoparticles	-	200–500 nm	-	- Induce selectively calcein dye releases from liposomes with embedded gold nanoparticles	[40]

**Table 2:** Optimization of graphene oxide or graphene oxide-poly (L-lysine) layer by layer liposome system [44].

Number of Layers	GO or GO-PLL to Liposome Mass Ratio	Size (nm)	Polydispersity Index	ζ Potential (mV)
One	1:2	153.9	0.12	−32.6 ± 4.8
Two	1:2	Aggregate	Aggregate	−9.8 ± 7.3
Two	2:1	243.8	0.1	39.9 ± 5.7
Three	2:1	170.7	0.17	−30.8 ± 7.5
Four	16:1	235.4	0.19	34.8 ± 5.9
Four	32:1	267.9	0.173	43.9 ± 6.9

**Table 3:** Non-targeted nanohybrid liposomal-metal systems.

Nanosystem	Drug	Most Relevant Effect	Ref.
Multiwalled carbon nanotubes (MWNTs) conjugated with cationic liposomes	Small interfering Polo-like kinase 1 (Si-PLK1) and doxorubicin (Dox)	- Cellular uptake in human A549-Luc cells was significantly higher by 3 folds as compared to L/siRNA carrier alone. - 40.3 ± 1.5% apoptotic cells in the nanohybrid system compared to 26.0 ± 4.3% in liposomes alone.	[98]
Titanium dioxide nanotubes (TNT)	5-Fluorouracil	- Sustained release of 5-FU for 5 weeks inhibitory concentration of 5-FU decreased from 470 μg/mL to 250 μg/mL upon loading into TNTs	[99]
Titanium dioxide zinc sulfide (TiO2 ZnS) nanotubes	5-Fluorouracil	- Initial lag phase for 1 h followed by a slow release.	[100]
Multiwalled carbon nanotubes	5-Fluorouracil	- Tumor decreased to half size upon combination.	[101]
